# Epidemiology and clinical characteristics of pathogens positive in hospitalized children with segmental/lobar pattern pneumonia

**DOI:** 10.1186/s12879-020-4938-7

**Published:** 2020-03-06

**Authors:** Yanxia Wang, Liji Ma, Ying Li, Yuyun Li, Yanfei Zheng, Xiaoyue Zhang

**Affiliations:** 1 Pediatrics, Zibo Central Hospital, No. 54, Gongqingtuanxi Street, Zibo City, 255036 Shandong Province China; 2 Allergic Clinic, Zibo Central Hospital, No.54, Gongqingtuanxi Street, Zibo City, 255036 Shandong Province China

**Keywords:** Epidemiology, Clinical characteristics, Pathogen, Segmental/lobar pattern pneumonia, Mycoplasma pneumoniae

## Abstract

**Background:**

The occurrence of segmental/lobar pattern pneumonia (S/L-PP) in children has recently increased. The pathogens of the disease may change for the misuse of antibiotics and the application of vaccines. Therefore, pathogens positive in hospitalized children with S/L-PP and their association with clinical characteristics may have changed. The aim of this study was to analyze the pathogens positive in hospitalized children with S/L-PP and their association with clinical characteristics.

**Method:**

The current study analyzed the epidemiological and clinical characteristics of pathogens positive in children with S/L-PP under 14 years old at a single hospital between 1st Jan 2014 and 31st Dec 2018 retrospectively. The pathogens were detected by microbial cultivation, indirect immunofluorescence of the kit (PNEUMOSLIDE IgM), Elisa, and/or real-time PCR in the samples of the patients.

**Results:**

A total of 593 children with S/L-PP received treatment at a single hospital during the study period by inclusion criteria. Four hundred fifty-one patients were single positive for one pathogen and 83 patients were positive for at least 2 pathogens. Mycoplasma pneumoniae (M.pneumoniae) (72.34%) was the most commonly detected pathogen, followed by *Streptococcus pneumoniae* (S.pneumoniae) (8.77%). The prevalence of M.pneumoniae in children with S/L-PP increased with time (*p* < 0.05). The positive rate of M.pneumoniae increased with ages of patients (*p* < 0.05). M.pneumoniae was statistically associated with the extrapulmonary manifestations while S.pneumoniae was statistically associated with abnormal white blood cells (WBCs) and C reactive proteins (CRPs) (*p* < 0.05).

**Conclusion:**

M.pneumoniae was the most positive pathogen in children with S/L-PP. The positive rate of M.pneumoniae in children with S/L-PP increased with time and the ages of children. M.pneumoniae was associated with extrapulmonary manifestations while S.pneumoniae was associated with abnormal WBCs and CRPs.

## Background

Community-acquired pneumonia (CAP) is one of the most common respiratory disorders in children, which often needs hospitalization [1]. Segmental/lobar pattern pneumonia (S/L-PP) is one of the common CAPs in children based on chest radiological findings of consolidation. Patients with S/L-PP often suffer from cough, fever, and even serious complications such as pulmonary atelectasis, pulmonary consolidation, pulmonary necrosis and respiratory failure, increasing the rate of morbidity, mortality as well as the cost of health care. The pathogens positive in children with S/L-PP may vary with regions, times, antibiotics use, and vaccines. The detection of pathogens often needs several hours or even days. In usual, pediatricians treat patients with antibiotics on experiences. The misuse of antibiotics may prolong the course of the disease, the suffering of patients and even cause more sequelaes. So it was important to find the pathogens positive in children with S/L-PP and their associations with clinical characteristics.

The occurrence of S/L-PP in children has increased with time clinically and has drawn the great attention of parents and pediatricians. In this research, the pathogens positive in children with S/L-PP and their clinical characteristics were retrospectively analyzed in hospitalized children who were admitted to Zibo Central Hospital during 1st Jan 2014 and 31st Dec 2018 as follows.

## Methods

### Patients

Zibo Central Hospital is situated in the central of Shandong Province in China. The hospital serves as a primary source of healthcare for people in Zibo area, which provides about six million people with common economic development and stable infrastructure. In the study, the medical records of children with pneumonia (as defined by the specifications in the International Classification of Diseases, 10th edition, ICD-10 code) who were admitted to Zibo Central Hospital between 1st Jan 2014 and 31st Dec 2018 were retrospectively analyzed.

The pneumonia pattern was characterized according to the World Health Organization Standardization of Interpretation of Chest Radiographs for the diagnosis of CAP in children [2]. Patients were included in the research by the inclusion criteria: 1) Patients with a chest radiograph performed during hospitalization; 2) Patients having a serological test of pathogens detected ≥7 days following the onset of the disease. Patients were excluded from the research according to the exclusion criteria: 1) Patients > 14 years of age; 2) Patients suffering from known coexisting chronic, progressive or oncological illnesses; 3) Patients with a chest radiograph of pulmonary perihilar linear opacities or reticulonodular infiltrates.

During the study period, a total of 9342 patients were admitted to the hospital and 593 patients with S/L-PP were included in this study. Data including genders, ages, clinical signs and symptoms, complications, laboratory and radiological findings, and durations of hospitalization of the patients were collected. Microbial cultivation was carried out by culturing and processing with blood or sputum specimens in accordance with standard microbiological procedures. Indirect immunofluorescence of the kit (PNEUMOSLIDE IgM) was used to detect the IgM antibodies against M. pneumoniae, respiratory syncytial virus (RSV), chlamydia pneumonia (CP), influenza A virus (IFA), parainfluenza virus (PIVS), adenovirus (ADV), Q fever Coxiella (COX), *Legionella pneumophila* (LP), and influenza B virus (IFB) according to the instructions. Specific IgM antibodies against M. pneumoniae were also assayed in sera samples from patients by ELISA. Realtime PCR was used to dectect M. pneumoniae and *Mycobacterium tuberculosis* in the bronchoalveolar lavage fluids of the patients. The patient was determined as pathogen positive if the pathogen was identified by any of the method.

### Statistical analysis

The Statistical package for the Social Science for Windows version 11.5 (SPSS, Inc., Chicago, IL, USA) was used for Statistical analyses. Continuous variables were expressed as mean ± standard deviation. For the age of patient may relate to the levels of certain laboratory indices such as erythrocyte sedimentation rate (ESR), white blood cell counts (WBCs) and C-reactive protein (CRP), they were transformed into categorical data (normal or abnormal). Poisson regression was used to evaluate the pathogens distribution over the years and seasons. Other categorical variables were assessed by the Chi-square test while the continuous variables were assessed by the method of t-test. *P* < 0.05 was indicated as a statistically significant difference.

## Results

### Overview of patients

Of 9342 children hospitalized with pneumonia (1752, 1803, 1849, 1885, and 2053) from 1st Jan 2014 to 31st Dec 2018, 593 patients with S/L-PP were enrolled in this study. Among them 398 patients were boys and the rest were girls. The male to female ratio was about 2:1. The age of the patients with S/L-PP ranged from 1 year to 13 years (7.4 ± 3.1 years). The number of patients with S/L-PP each year was 86, 98,115,137,157 respectively from 2014 to 2018. The annual incidence of S/L-PP increased with time over the study period (*P* < 0.05). The durations of fever and cough were 4.6 ± 2.1 days and 10.6 ± 8.7 days respectively. One hundred sixty-nine patients had a gasping and 208 patients had pulmonary crackles at onset. There were 149 patients with extrapulmonary manifestations including 126 cases with erythematous maculopapular rash, 69 cases with liver function lesions, 5 cases with kidney function lesions, and 51 cases with neurological complications (2 cases were diagnosed as autoimmune encephalitis). Only a few patients had pleural effusion. There were 383 patients with abnormal WBCs, 69 patients with abnormal ESRs and 148 patients with abnormal CRPs. The average duration of hospital stay was 15.5 ± 3.1 days.

### Pathogens distribution with time

Table 1 showed the distribution of pathogens with time including M. pneumoniae, RSV, CP, IFA, PIVS, ADV, COX, LP, IFB, S.pneumoniae, *Staphylococcus aureus* (*S. aureus*), *Pseudomonas aeruginosa* (P.aeruginosa), *Escherichia coli* (E.coli) and *Klebsiella pneumoniae* (K.pneumoniae), and showed the positive rate of M.pneumoniae increased with time. The number of patients positive with M.pneumoniae was 43, 67, 96,106, and 117 respectively each year during the study period, and the positive rate of M.pneumoniae between the groups with time was significantly different (*p* < 0.05). But no significant differences in the positive rate for other pathogens with years between the groups were found.

**Table 1:** Pathogen distribution with time in patients with segmental/lobar pattern pneumonia (S/L-PP)

Year		2014	2015	2016	2017	2018	*p*
pneumoniae		1752	1803	1845	1885	2053	
S/L-PP		86 (4.91%)	98 (5.44%)	115 (6.23%)	137 (7.27%)	157 (7.65%)	
M.pneumoniae	pneumoniae	451 (25.74%)	452 (23.57%)	523 (28.35%)	594 (31.51%)	734 (35.75%)	
	S/L-PP	43 (50.00%)	67 (68.37%)	96 (83.48%)	106 (77.37%)	117 (74.52%)	< 0.01
RSV	pneumoniae	165 (9.42%)	205 (11.37%)	209 (11.33%)	215 (11.41%)	235 (11.45%)	
	S/L-PP	5 (5.81%)	4 (4.08%)	5 (4.35%)	3 (2.19%)	3 (1.91%)	> 0.05
CP	pneumoniae	32 (1.83%)	49 (2.72%)	61 (3.31%)	36 (1.91%)	52 (2.53%)	
	S/L-PP	4 (4.65%)	4 (4.08%)	4 (3.48%)	2 (1.46%)	2 (1.27%)	> 0.05
IFA	pneumoniae	99 (5.65%)	131 (7.27%)	80 (4.34%)	116 (6.15%)	152 (7.40%)	
	S/L-PP	4 (4.65%)	1 (1.02%)	3 (2.61%)	3 (2.19%)	2 (1.27%)	> 0.05
PIVS	pneumoniae	58 (3.31%)	61 (3.38%)	42 (2.28%)	98 (5.20%)	105 (5.11%)	
	S/L-PP	6 (6.98%)	5 (5.10%)	5 (4.35%)	6 (4.38%)	4 (2.55%)	> 0.05
ADV	pneumoniae	169 (9.65%)	185 (10.26%)	145 (7.86%)	220 (11.67%)	173 (8.43%)	
	S/L-PP	5 (5.81%)	5 (5.10%)	4 (3.48%)	4 (2.92%)	2 (1.27%)	> 0.05
COX	pneumoniae	98 (5.59%)	73 (4.05%)	84 (4.55%)	79 (4.19%)	112 (5.46%)	
	S/L-PP	4 (4.65%)	5 (5.10%)	5 (4.35%)	4 (2.92%)	4 (2.55%)	> 0.05
LP	pneumoniae	39 (2.23%)	45 (2.50%)	53 (2.87%)	71 (3.77%)	46 (2.24%)	
	S/L-PP	3 (3.49%)	3 (3.06%)	4 (3.48%)	1 (0.73%)	1 (0.64%)	> 0.05
IFB	pneumoniae	102 (5.82%)	134 (7.42%)	163 (8.83%)	218 (11.56%)	213 (10.38%)	
	S/L-PP	4 (4.65%)	4 (4.08%)	2 (1.74%)	4 (2.92%)	3 (1.91%)	> 0.05
S.pneumoniae	pneumoniae	85 (4.85%)	74 (4.10%)	90 (4.88%)	88 (4.67%)	101 (4.92%)	
	S/L-PP	11 (12.79%)	10 (10.20%)	10 (8.70%)	11 (8.03%)	10 (6.37%)	> 0.05
*S. aureus*	pneumoniae	4 (0.23%)	5 (0.28%)	4 (0.22%)	3 (0.16%)	4 (0.19%)	> 0.05
	S/L-PP	0 (0.00%)	0 (0.00%)	2 (1.74%)	0 (0.00%)	0 (0.00%)	
P.aeruginosa	pneumoniae	2 (0.11%)	2 (0.11%)	1 (0.05%)	2 (0.11%)	1 (0.05%)	> 0.05
	S/L-PP	0 (0.00%)	0 (0.00%)	0 (0.00%)	1 (0.73%)	0 (0.00%)	
*E. coli*	pneumoniae	10 (0.57%)	8 (0.44%)	9 (0.49%)	7 (0.37%)	5 (0.24%)	> 0.05
	S/L-PP	1 (1.16%)	0 (0.00%)	1 (0.87%)	1 (0.73%)	0 (0.00%)	
K.pneumoniae	pneumoniae	2 (0.11%)	5 (0.28%)	4 (0.22%)	3 (0.16%)	2 (0.10%	
	S/L-PP	0 (0.00%)	0 (0.00%)	0 (0.00%)	1 (0.73%)	0 (0.00%)	> 0.05

### Age distribution of pathogens

Table 2 summarized the distribution of pathogens with age groups and showed that the positive rate of M.pneumoniae increased with ages. Significant differences were observed in the positive rate of M.pneumoniae between the age groups. However, no significant differences were found in the positive rate of other pathogens between the age groups.

**Table 2:** Age distribution of pathogens in patients with S/L-PP

Age	Age < 6 year	6 ≤ age<14	χ^2^	*p*
n	189	404		
M.pneumoniae	112 (59.26%)	317 (78.47%)	25.74	< 0.01
RSV	9 (4.76%)	11 (2.72%)	1.64	> 0.05
CP	7 (3.70%)	9 (2.23%)	1.07	> 0.05
IFA	4 (2.12%)	9 (2.23%)	0.05	> 0.05
PIVS	7 (3.70%)	19 (4.70%)	0.31	> 0.05
ADV	8 (4.23%)	11 (2.72%)	0.95	> 0.05
COX	6 (3.17%)	17 (4.21%)	0.37	> 0.05
LP	5 (2.65%)	7 (1.73%)	0.18	> 0.05
IFB	8 (4.23%)	10 (2.48%)	1.35	> 0.05
S.pneumoniae	16 (8.47%)	36 (8.91%)	0.03	> 0.05

### Sex distribution of pathogens

Significant differences were not observed for M. pneumoniae and S.pneumoniae between male patients and female patients. Eighteen patients were positive for IFB including 6 male patients and 12 female patients. Female patients displayed significantly higher positive rate for IFB. No significant sex difference was observed for the other pathogens.

### Season distribution of pathogens

In general, the seasonality profile of each individual pathogen was diverse. However, we did not observe a distinct pattern for the pathogens.

### Mixed infection types of pathogens

Co-infections with multiple pathogens were common. There were 91 patients in whom 2 or more pathogens were positive, representing 15.34% of the patients, and the types of co-infection were complex. These data indicated that 27.40% of the children with M.pneumoniae infections were co-infected with other pathogens. A total of 15 patients showed infection with 3 pathogens or more (Table 3).

**Table 3:** Mixed infection types of pathogens

Co-infection type	Number
2 pathogens	76
M.pneumoniae +RSV	5
M.pneumoniae +CP	4
M.pneumoniae +IFA	4
M.pneumoniae +PIVS	7
M.pneumoniae +ADV	4
M.pneumoniae +COX	10
M.pneumoniae +LP	4
M.pneumoniae +IFB	6
M.pneumoniae + S.pneumoniae	20
M.pneumoniae + S. aureus	2
M.pneumoniae + K.pneumoniae	1
M.pneumoniae + E.coli	1
RSV + CP	1
RSV+ E.coli	1
CP + IFA	1
CP + PIVS	1
CP + ADV	1
CP+ S.pneumoniae	1
IFA + LP	1
COX + LP	1
3	14
M.pneumoniae +CP + ADV	1
RSV + LP + IFB	1
PIVS+ADV + COX	1
M.pneumoniae +PIVS+ADV	1
M.pneumoniae +CP+ S.pneumoniae	1
M.pneumoniae +RSV + LP	1
M.pneumoniae +CP + IFA	1
M.pneumoniae +ADV + IFB	1
M.pneumoniae +PIVS+COX	1
M.pneumoniae +LP+ S.pneumoniae	1
M.pneumoniae +IFA+ P.aeruginosa	1
M.pneumoniae +ADV + COX	1
M.pneumoniae +RSV + CP	1
M.pneumoniae +IFA + COX	1
4	1
M.pneumoniae +IFA + ADV + COX	1

### Association between pathogens and patients’ demographic and clinical characteristics

Table 4 summarized the patients’ demographic and clinical information found in association with pathogen infections. The patients were divided into groups according to pathogens. Patients positive with 2 pathogens or more were excluded. Since the sample size was too small to obtain significance in some statistical analyses, only M. pneumoniae and S.pneumoniae were included in the statistical analyses. M.pneumoniae was statistically associated with the extrapulmonary manifestations. S.pneumoniae was statistically associated with abnormal WBCs and CRPs (Table 5).

**Table 4:** Association between pathogens and patients’ demographic and clinical characteristics

Variables	M.pneumoniae	RSV	CP	IFA	PIVS	ADV	COX	LP	IFB	S.pneumoniae
N	353	11	7	3	14	8	6	4	8	28
Gender										
male	246 (69.69%)	10 (90.91%)	3 (42.86%)	3 (100.00%)	10 (71.43%)	4 (50.00%)	3 (50.00%)	2 (50.00%)	4 (50.00%)	20 (71.43%)
female	107 (30.31%)	1 (9.09%)	4 (57.14%)	0 (0.00%)	4 (28.57%)	4 (50.00%)	3 (50.00%)	2 (50.00%)	4 (50.00%)	8 (28.57%)
Age (years)	7.8 ± 4.1	8.4 ± 3.1	10.2 ± 2.6	5.4 ± 3.6	6.5 ± 5.2	6.8 ± 4.5	7.6 ± 3.8	8.3 ± 5.2	6.8 ± 3.9	7.9 ± 3.5
Fever										
yes	302 (85.55%)	8 (72.73%)	5 (71.43%)	3 (100.00%)	10 (71.43%)	7 (87.50%)	4 (66.67%)	3 (75.00%)	6 (75.00%)	21 (75.00%)
no	51 (14.45%)	3 (27.27%)	2 (28.57%)	0 (0.00%)	4 (28.57%)	1 (12.50%)	2 (33.33%)	1 (25.00%)	2 (25.00%)	7 (25.00%)
Duration of fever (days)	4.9 ± 2.8	5.7 ± 3.2	3.5 ± 2.6	4.3 ± 3.2	3.8 ± 2.3	4.5 ± 1.9	5.6 ± 2.4	4.1 ± 2.6	4.7 ± 2.6	4.5 ± 2.4
Duration of cough (days)	10.2 ± 6.2	8.6 ± 5.8	13.6 ± 6.5	10.3 ± 6.9	11.8 ± 9.3	8.9 ± 4.3	10.1 ± 6.8	8.2 ± 4.3	9.4 ± 7.6	11.3 ± 6.4
Gasping										
Yes	122 (34.56%)	3 (27.27%)	0 (0.00%)	0 (0.00%)	1 (7.14%)	2 (25.00%)	0 (0.00%)	0 (0.00%)	0 (0.00%)	2 (7.14%)
No	231 (65.44%)	8 (72.73%)	7 (100.00%)	3 (100.00%)	13 (92.86%)	6 (75.00%)	6 (100.00%)	4 (100.00%)	8 (100.00%)	26 (92.86%)
Pulmonary crackles at onset										
yes	120 (33.99%)	3 (27.27%)	2 (28.57%)	0 (0.00%)	4 (28.57%)	2 (25.00%)	2 (33.33%)	1 (25.00%)	3 (37.50%)	9 (32.14%)
no	233 (66.01%)	8 (72.73%)	5 (71.43%)	3 (100.00%)	10 (71.43%)	6 (75.00%)	4 (66.67%)	3 (75.00%)	5 (62.50%)	19 (67.86%)
Pleural effusion										
Yes	15 (4.25%)	2 (18.18%)	1 (14.29%)	0 (0.00%)	1 (7.14%)	0 (0.00%)	0 (0.00%)	0 (0.00%)	1 (12.50%)	1 (3.70%)
no	340 (96.32%)	9 (81.82%)	6 (85.71%)	3 (100.00%)	13 (92.86%)	8 (100.00%)	6 (100.00%)	4 (100.00%)	7 (87.50%)	27 (96.43%)
Extrapulmonary manifestations										
Yes	102 (28.90%)	0 (0.00%)	0 (0.00%)	1 (33.33%)	2 (14.29%)	1 (12.50%)	0 (0.00%)	0 (0.00%)	1 (12.50%)	3 (10.71%)
Erythematous maculopapular rash	20 (5.67%)	0 (0.00%)	0 (0.00%)	0 (0.00%)	0 (0.00%)	0 (0.00%)	0 (0.00%)	0 (0.00%)	0 (0.00%)	2 (7.14%)
Liver lesion	46 (13.03%)	0 (0.00%)	0 (0.00%)	0 (0.00%)	0 (0.00%)	0 (0.00%)	0 (0.00%)	0 (0.00%)	0 (0.00%)	0 (0.00%)
Kidney lesion	9 (2.55%)	0 (0.00%)	0 (0.00%)	0 (0.00%)	0 (0.00%)	0 (0.00%)	0 (0.00%)	0 (0.00%)	0 (0.00%)	0 (0.00%)
Neurological complications	79 (22.38%)	0 (0.00%)	0 (0.00%)	1 (33.33%)	2 (14.29%)	1 (12.50%)	0 (0.00%)	0 (0.00%)	1 (12.50%)	1 (3.57%)
no	251 (71.10%)	11 (100.00%)	7 (100.00%)	2 (66.67%)	12 (85.71%)	7 (87.50%)	6 (100.00%)	4 (100.00%)	7 (87.50%)	25 (89.29%)
WBC										
abnormal	245 (69.41%)	5 (45.45%)	4 (57.14%)	2 (66.67%)	6 (42.86%)	5 (62.50%)	2 (33.33%)	2 (50.00%)	4 (50.00%)	27 (96.43%)
normal	108 (30.59%)	6 (54.55%)	3 (42.86%)	1 (33.33%)	8 (57.14%)	3 (37.50%)	4 (66.67%)	2 (50.00%)	4 (50.00%)	1 (3.57%)
ESR										
abnormal	36 (10.20%)	1 (9.09%)	2 (28.57%)	0 (0.00%)	1 (7.14%)	1 (12.50%)	0 (0.00%)	1 (25.00%)	2 (25.00%)	3 (10.71%)
normal	317 (89.80%)	10 (90.91%)	5 (71.43%)	3 (100.00%)	13 (92.86%)	7 (87.50%)	6 (100.00%)	3 (75.00%)	6 (75.00%)	25 (89.29%)
CRP										
abnormal	81 (22.95%)	3 (27.27%)	2 (28.57%)	1 (33.33%)	4 (28.57%)	3 (37.50%)	2 (33.33%)	1 (25.00%)	3 (37.50%)	24 (85.71%)
normal	272 (77.05%)	8 (72.73%)	5 (71.43%)	2 (66.67%)	10 (71.43%)	5 (62.50%)	4 (66.67%)	3 (75.00%)	5 (62.50%)	4 (14.29%)
Duration of hospitalization (days)	15.8 ± 4.1	14.2 ± 4.3	13.6 ± 5. 8	12.5 ± 3.6	14.9 ± 5.2	15.1 ± 3.7	13.9 ± 6.2	14.7 ± 5.1	14.6 ± 2.4	15.3 ± 4.4

**Table 5:** Comparison between M.pneumoniae and S.pneumoniae with patients’ demographic and clinical characteristics

Variables	M.pneumoniae	S.pneumoniae	χ^2^	*p*
N	353	28		
Gender				
male	246 (69.69%)	20 (71.43%)	2.06	> 0.05
female	107 (30.31%)	8 (28.57%)		
Age (years)	7.8 ± 4.1	7.9 ± 3.5	0.13	> 0.05
Fever				
yes	302 (85.55%)	21 (75.00%)		
no	51 (14.45%)	7 (25.00%)	1.5	> 0.05
Duration of fever (days)	4.9 ± 2.8	4.5 ± 2.4	0.73	> 0.05
Duration of cough (days)	10.2 ± 6.2	11.3 ± 6.4	0.90	> 0.05
Gasping				
Yes	122 (34.56%)	2 (7.14%)		
No	231 (65.44%)	26 (92.86%)	8.88	< 0.01
Pulmonary crackles at onset				
yes	120 (33.99%)	9 (32.14%)		
no	233	19 (67.86%)	0.05	> 0.05
Pleural effusion				
Yes	15 (4.25%)	1 (3.70%)		
no	340 (96.32%)	27 (96.43%)	0.03	> 0.05
Extrapulmonary manifestations				
Yes	102 (28.90%)	3 (10.71%)		
Erythematous maculopapular rash	20 (5.67%)	2 (7.14%)		
Liver lesion	46 (13.03%)	0 (0.00%)		
Kidney lesion	9 (2.55%)	0 (0.00%)		
Neurological complications	79 (22.38%)	1 (3.70%)		
no	251 (71.10%)	25 (89.29%)	10.72	< 0.05
WBC				
abnormal	245 (69.41%)	27 (96.43%)		
normal	108 (30.59%)	1 (3.70%)	9.28	< 0.01
ESR				
abnormal	36 (10.20%)	3		
normal	317 (89.80%)	25 (89.29%)	0.06	> 0.05
CRP				
abnormal	81 (22.95%)	24 (85.71%)		
normal	272 (77.05%)	4 (14.29%)	51.2	< 0.01
Duration of hospitalization (days)	15.8 ± 4.1	15.3 ± 4.4	0.62	> 0.05

## Discussion

S/L-PP is a common pediatric low respiratory tract infection [3], which is involved in the community-acquired pneumonias (CAP). The incidence of S/L-PP has recently increased in clinical practice. The considerably serious clinical manifestations including hyperpyrexia, cough and expiratory dyspnea often result in extra pulmonary multi-system complications. Currently there were no standardized therapeutic strategies on pediatric S/L-PP [3]. Although new antibiotics are developed increasingly, no obvious fall in the morbidity and mortality of S/L-PP has been observed. Generally, patients with S/L-PP often have more severe symptoms than those with no S/L-PP. S/L-PP was more closely related to severe manifestations, including pleural effusion, higher rates of fever, extrapulmonary manifestations, abnormal WBCs, abnormal CRPs and bacterial co-infection, as well as longer durations of fever and hospitalization [4]. In our research, the duration of fever and hospitalization of the patients with S/L-PP were 4.6 ± 2.1 days and 15.5 ± 3.1 days, which were similar to the previous report [4]. However, the pathogens positive in the patients with S/L-PP and their association with clinical characteristics in children has not been ever found to be reported. The microbes are difficult to isolate in children with S/L-PP for the difficulties in sputum expectoration and low positive rate of blood culture [5]. Some detection may be positive about a week after the onset of the disease. Therefore, the treatment of the disease based on knowledge and experience is very important. This research described the pathogens and their association with clinical characteristics in the patients with S/L-PP, which could add knowledge and experience of the disease for clinical doctors to treat it.

The positive rate of the pathogens in patients with S/L-PP was highly diverse in this research. M. pneumoniae was the most commonly detected pathogen. The total positive rate of M. pneumoniae was 72.34% (429/593) and increased with time, which suggested M. pneumoniae was highly associated with the disease. This was different from the previous report [6, 7]. In fact, it was estimated that M. pneumoniae infection was accountable for up to 30–40% of CAP [8–11]. The classical radiological manifestations of M. pneumoniae pneumonia included segmental/lobar air-space consolidation, diffuse tiny centrilobular nodules and bronchovascular thickening [12–15]. The S/L-PP was considered to account for 17–76.5% of pediatric M. pneumoniae pneumonia cases. The incidence of S/L-PP showed an increasing trend [16–19]. So M. pneumoniae had drawn the great attention of clinical doctors and patients. However, there had been no any type of vaccines approved for use against M. pneumoniae [20]. The positive rate of M. pneumoniae in patients with S/L-PP increased with ages of children. It was postulated with 2 explanations. First, old patients preferred social activity in herd and chances for them to be infected were high. Second, the progression of the immune system in the patients was different between old patients and young ones. A report suggested that M. pneumoniae pneumonia was closely correlated with the immune system of the patients [20]. The different progression state of the immune system between old patients and young ones may be related with the different positive rate of M. pneumoniae in the patients. The positive rate of M. pneumoniae in male patients was not statistically different from female ones, which suggested that M. pneumoniae infection was not affected by sex ratio. The patients with S/L-PP infected by M. pneumoniae occurred all the year round and didn’t vary with the changes of seasons. The extrapulmonary complications in patients with S/L-PP infected by M. pneumoniae were common and the prevalence of this kind of complication may be up to 26.17% [4], which was similar to the results in this research. However the complications occurred few in patients positive with other pathogens and was not discussed in the research.

The second positive rate of pathogen in patients with S/L-PP was S.pneumoniae and it was 8% in the research. The positive rate of S.pneumoniae was much lower than that of M. pneumoniae, which was different from the previous understanding [6, 7]. It may be associated with the application of S.pneumoniae vaccines in China, which could stop S.pneumoniae infection [21–24]. The misuse of antibiotics was common in the nationwide, which could cause the low positive rate of S.pneumoniae. Microbial cultivation could bring false negative results in some samples. Samples were usually taken after the patients having taken oral or intravenous antibiotics. It was another reason for the low positive rate of S.pneumoniae in the study. Compared with other pathogens, S.pneumoniae was significantly associated with abnormal WBCs and CRPs, which may be used for the determination of S/L-PP pathogens in clinical practice. However, M.pneumoniae and S.pneumoniae in children with S/L-PP counted for 81.1% of the pathogens in total, which was much higher than that reported by Saraya T [25]. Other pathogens had low positive rate in this research, which was not discussed here.

Some patients were positive with two or more pathogens in the research. Two pathogens positive were the most common. The common two pathogens positive were M. pneumoniae and S.pneumoniae. 3 pathogens or more positive were less. The association between 2 pathogens or more and their clinical characteristics were not further discussed here for small cases.

The study was also associated with some limitations. First, clinical data were collected from medical records retrospectively, and there may have been some selection bias. Second, it was regretful that almost all the pathogens were detected by single specific IgMs for parents of the patients didn’t allow another detection of IgMs in a few weeks later. Therefore, the patients were denoted as “positive” rather than “confirmed” by one pathogen infection. These results could still provide help for doctors in primary hospitals in China. Third, the sample size of some samples was not large enough to obtain significance in some statistical analyses. In addition, some pathogens may not be found due to the limitation of the detection method.

## Conclusion

In a summary, M. pneumoniae was the most frequent detected pathogen in the children with S/L-PP. The prevalence of M. pneumoniae infection increased with time and ages of children. Old patients were more prone to be infected by M. pneumoniae. M. pneumoniae was associated with extrapulmonary manifestation while S.pneumoniae was associated with abnormal WBCs and CRPs.

## Data Availability

The datasets used and/or analyzed during the current study are available from the corresponding author on reasonable request.

## Abbrevations

ADV: Adenovirus
CAP: Community-acquired pneumonia
COX: Q fever Coxiella
CP: Chlamydia pneumonia
CRPs: C reactive proteins
*E. coli*: *Escherichia coli*
ESR: Erythrocyte sedimentation rate
IFA: Influenza A virus
IFB: Influenza B virus
K.pneumoniae: *Klebsiella pneumoniae*
LP: *Legionella pneumophila*
M.pneumoniae: Mycoplasma pneumoniae
P.aeruginosa: *Pseudomonas aeruginosa*
PIVS: Parainfluenza virus
RSV: Respiratory syncytial virus
*S. aureus*: *Staphylococcus aureus*
S.pneumoniae: *Streptococcus pneumoniae*
S/L-PP: Segmental/lobar pattern pneumonia
WBCs: White blood cells
